# Efficacy of Functional Foods, Beverages, and Supplements Claiming to Alleviate Air Travel Symptoms: Protocol for a Systematic Review

**DOI:** 10.2196/16155

**Published:** 2020-03-27

**Authors:** Virginia Chan, Margaret Allman-Farinelli

**Affiliations:** 1 Nutrition and Dietetics Group School of Life and Environmental Science The University of Sydney Camperdown Australia

**Keywords:** aircraft, dietary supplements, functional food, functional beverage, jetlag syndrome, sleep

## Abstract

**Background:**

Airline passengers often experience symptoms when travelling on long and ultra-long flights. These range from minor discomforts such as gastrointestinal symptoms to more serious life-threatening clinical conditions such as deep vein thrombosis. The food and supplement industry have responded with a plethora of products that claim to prevent one or more of the physiological or psychological symptoms associated with air travel.

**Objective:**

The aim of this literature review is to evaluate the efficacy of functional foods, beverages, and supplements that claim to address the unwanted effects of air travel in healthy adult populations.

**Methods:**

This research is a two-stage process. The first step is a scoping review of the functional foods, beverages, and supplements making claims that they lessen or prevent the physical or psychological symptoms associated with commercial air travel. Databases (ie, Medline, Embase, PsycINFO, and Web of Science), gray literature (ie, the flight catering magazines PAX International, APEX, and Onboard Hospitality), and search engines (ie, Google and Bing) will be used to identify products and generate a database. The second stage is a systematic literature review of the evidence supporting any health claims made for such products. The search will be conducted in Medline, Embase, PsycINFO, Cumulative Index to Nursing and Allied Health Literature, and Cochrane Central Register of Controlled Trials. Additionally, gray literature that includes the reference list of studies included in the systematic literature review and scientific articles referenced by the products within our database will be hand searched. Randomized and nonrandomized controlled trials reporting on changes in flight-related physical or cognitive symptoms in healthy adults that were conducted in commercial flight or flight simulation settings will be included. Two authors will independently screen, extract data, and assess the strength of evidence and risk of bias of the studies. The strength of evidence will be judged using the Grading of Recommendations, Assessments, Developments, and Evaluations approach, and the risk of bias will be assessed using the appropriate Cochrane Collaboration tool (Risk of Bias for Randomized Control Trials II or Robins I for Nonrandomized Interventions).

**Results:**

The scoping review of available functional foods, beverages, and supplements was conducted from March 6, 2019, to August 31, 2019. The systematic literature review commenced on October 1, 2019. The review is expected to be completed in 2020.

**Conclusions:**

The review findings will help consumers and employees of commercial airlines make informed decisions on their use of functional foods and beverages for alleviating air travel–related symptoms.

**International Registered Report Identifier (IRRID):**

DERR1-10.2196/16155

## Introduction

With the increasing popularity of international air travel, more people are exposed to extended flight conditions. Long (12-16 hours) and ultra-long (16+ hours) range flights [1] have been associated with several physiological and psychological symptoms that can affect both the air travelling public and commercial cabin crews.

Jetlag, the desynchronization of the normal circadian rhythm due to rapid travel through multiple time zones, is perhaps the most iconic condition associated with prolonged flight [2,3]. Typical symptoms of jetlag include fatigue, sleep disruption, hindered capability to perform cognitive and physical tasks, and mood disturbances [3,4]. The severity of these symptoms is related to the number of time zones crossed and the direction of travel, especially for eastward travel over multiple time zones [3-5].

Prolonged flight conditions can also produce physical symptoms. The average cabin air pressure at typical cruising altitudes results in a 35% expansion of gases within the gastrointestinal tract causing the sensation of abdominal distension and bloating [6,7]. Low cabin humidity combined with the diuretic effects of alcohol and caffeinated beverages commonly consumed preflight or in-flight contributes to dehydration [8], dry skin [9], and irritation of the mucosal membranes in the nose and throat [7]. Compromised mucosal membranes and the proximity of passengers may increase passenger susceptibility to upper respiratory tract infections [7,10]. The mildly hypoxic conditions in pressurized cabins combined with dehydration and reduced physical activity increases the risk of developing deep vein thrombosis, pulmonary embolism, and edema [7,8]. Of concern to cabin crews and frequent travelers, the high altitude of flights increases individual’s exposure to cosmic radiation and reactive oxygen species [11].

A number of pharmacological and nonpharmacological treatments exist to lessen the symptoms associated with flight, such as light therapy for jetlag [12] and high-efficiency particulate air filters to improve air quality [7,10]. In addition, the food and supplement industries have responded with a plethora of products claiming to reduce or relieve one or more of these physiological or psychological symptoms. These products include melatonin to improve sleep and reduce jetlag, caffeine to address fatigue, herbal extracts to improve immunity or reduce risk of thrombotic events, and various vitamins and minerals for hydration or as protection against DNA damage from cosmic radiation. However, companies may fail to provide evidence from in-flight settings or flight simulations to justify their claims, and an evidence-based approach is required to provide appropriate advice for passengers and produce guidelines for airlines.

This study aims to evaluate the efficacy of functional foods, beverages, and supplements that claim to alleviate the effects of air travel in healthy populations.

## Methods

### Study Design

**Figure 1:**
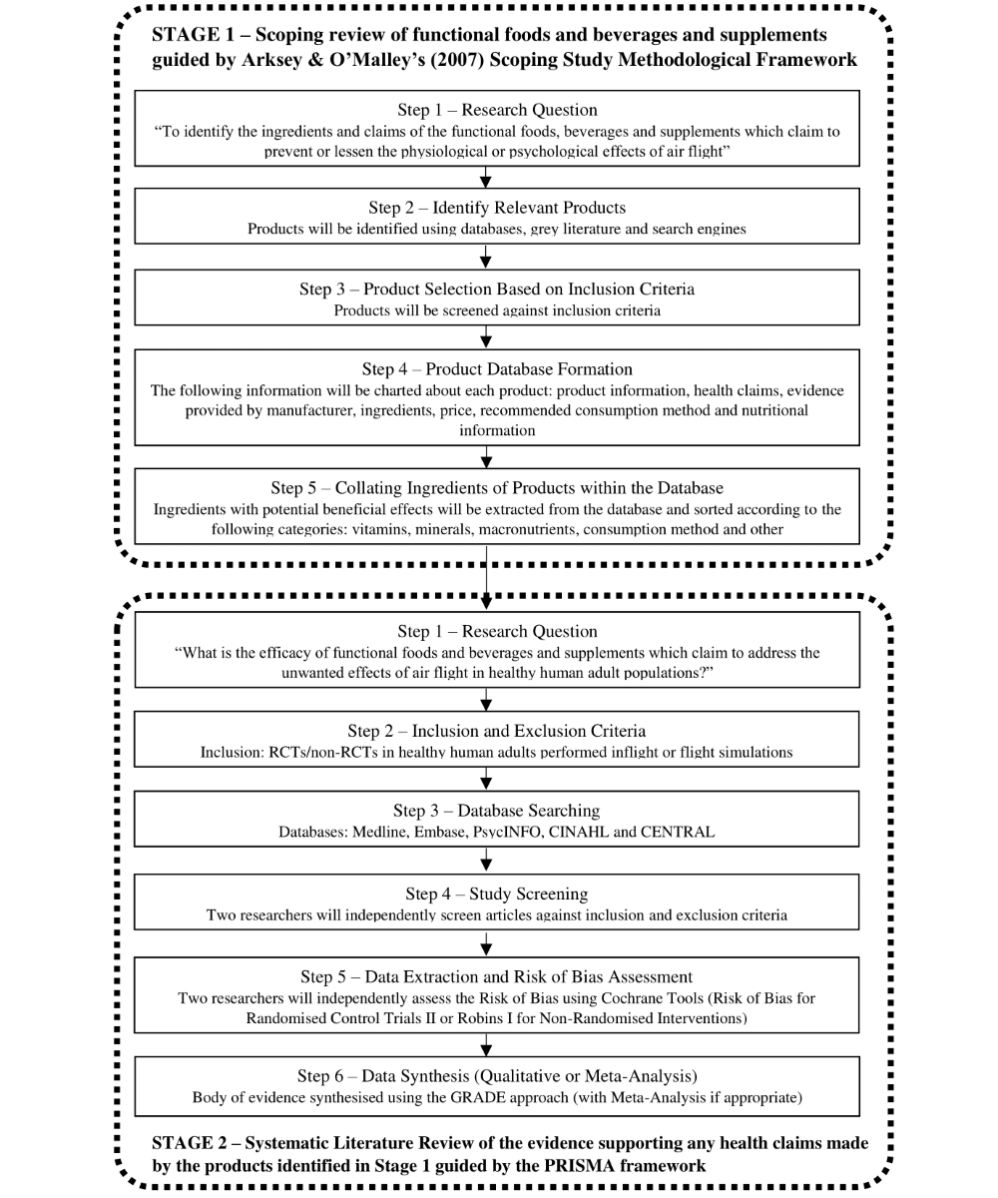
Flow diagram of study design. RCT: randomized controlled trial; GRADE: Grading of Recommendations, Assessment, Development and Evaluations; PRISMA: Preferred Reporting Items for Systematic Reviews and Meta-Analysis.

This study is a two-phase process outlined in Figure 1. The first phase is a scoping review of functional foods, beverages, and supplements that claim to alleviate the physical or cognitive symptoms associated with commercial air travel. This stage will be guided by Arksey & O’Malley’s (2007) Scoping Study Methodological Framework [13], and the identified products will form the database. The second stage is a systematic literature review of the evidence surrounding any health claims made by the products in the database. The PRISMA (Preferred Reporting Items for Systematic Review and Meta-Analyses) framework [14] will be used to direct the systematic review process and report the outcomes.

### Scoping Review

#### Scoping Review Search Strategy to Identify Relevant Products

The scoping review of available functional foods, beverages, and supplements will be conducted in databases (ie, Medline, Embase, PsycINFO, and Web of Science) and search engines (ie, Google and Bing). Well-known publicly available travel catering gray literature (ie, PAX International, APEX, and Onboard Hospitality) that includes articles and advertisements of relevant functional foods, beverages, and supplements will also be searched. Search terms will include combinations, truncations, and synonyms of: “drinks”, “beverages”, “food”, “snacks”, “nutrition”, or “supplements” combined with “airplane”, “aviation”, “deep vein thrombosis”, “flight”, “hydration”, “jetlag”, or “sleep”.

#### Product Inclusion and Exclusion Criteria

Products identified in the scoping review will be included in the database if they meet any one of the following inclusion criteria.

1. The product is stocked or marketed to airlines, airports, or commercial cabin crews.
2. The product is claimed to be used or developed by commercial cabin crews.
3. There is a scientific publication performing trials of the product in a flight setting or flight simulation.
4. The product is specifically designed for or indicates it can be used in commercial flight settings.

Products will be excluded from the database if they were discontinued at the time of the search.

#### Product Database Formation

The following information will be transcribed into Microsoft Excel 2011 (Microsoft, Redmond, WA).

1. Product information including: brand, product name, flavor, serving size, product type, country of origin, parent company, location of procurement, nutrient reference range, website, date of initial product entry, and date of latest entry update
2. Health claims classified into one or more of the following categories: jetlag, fatigue, sleep, cognitive ability, immunity, hydration status, cardiovascular, protection against radiation, inflammation, anxiety, or gastrointestinal symptoms
3. Evidence provided by the manufacturer supporting any claims made will be categorized as: scientific article in flight setting; scientific article not in flight setting; trial of product; food authority; academic research institute, government agency, or nongovernment organization; customer testimonies; medical or academic professionals; generalized statements without evidence; or other
4. Advertised ingredients and full ingredients list
5. Price per serve (AUD) at time of entry
6. Recommended method of consumption
7. Nutritional Information per serve and per 100 g

#### Collating Ingredients of Products Within the Database

Active ingredients of functional foods and nutrients will be extracted and sorted according to the following categories: vitamins, minerals, macronutrients, consumption method, and other. Ingredients with possible beneficial effects on symptoms relating to flight will be further examined by a systematic literature review.

### Systematic Literature Review

#### Search Terms

Search terms will be the ingredients of products within the database. These will be combined with synonyms and truncations of aviation terms such as air travel, aviation, cabin crew, and travel medicine. The Medline Thesaurus MeSH (Medical Subject Headings) term will be refined according to each database. The Scottish Intercollegiate Guidelines Network (SIGN) randomized controlled trial study filter [15] will be adapted to capture nonrandomized controlled trials and applied to searches conducted within Medline, Embase, and Cumulative Index to Nursing and Allied Health Literature (CINAHL).

#### Search Strategy

The databases CINAHL, Cochrane Central Register of Controlled Trials (CENTRAL), Embase, Medline (including PreMedline), and PsycINFO will be searched from inception for studies. Any published scientific articles referenced by products in the database and the reference lists of included studies will be hand searched for additional citations.

#### Study Eligibility Criteria

Eligibility criteria for studies have been selected based on PICOS (participants, interventions, comparisons, outcomes, and study designs) standards.

#### Types of Participants

The target age group for included studies is adults aged 18 years or older. No animal studies will be included. Participants should be healthy with no pre-existing health conditions that could impact the primary outcome of intervention, such as hematological abnormalities. Studies that involve pregnant women or participants aged 18 years or younger are not eligible for inclusion. There will be no limitations placed on gender or ethnicity.

#### Types of Interventions

Intervention must involve administration of a functional food, beverage, or nutritional supplement to participants at any time before, during, or after a commercial flight or flight simulation that is intended to improve the well-being of the participant. No limitations will be placed on length of intervention or follow-up period. Studies that solely involve the use of pharmacological agents (with the exception of melatonin and caffeine) or nonpharmacological treatments other than nutrition such as physical activity will be excluded. Interventions that use a combination of nonpharmacological treatments but also test functional foods, beverages, or nutritional supplements will be eligible for inclusion if the effect of the functional or nutritional products can be isolated.

#### Types of Comparisons

Studies must make a comparison between those who received intervention and those who did not receive intervention or were given a placebo, and both groups must have undergone the same in-flight or flight simulation.

#### Types of Outcomes Measured

The primary outcome of this systematic literature review is to determine if there is an improvement in the physical or cognitive symptoms associated with air travel between participants that received intervention and the controls. This can be reported using valid qualitative and quantitative measures.

The secondary outcomes that will be investigated are the incidence of toxicity or negative effects within the intervention or control groups, study funding sources, and the prevalence of industry funding.

#### Types of Study Designs

Studies will be limited to randomized and nonrandomized controlled trials conducted using in-flight or flight simulation settings. All other study types and non-English studies will be excluded. Studies completed under space or military flight conditions will also be excluded, as the conditions of speed and altitude are not comparable to commercial air travel.

#### Study Selection

Bibliographic records for all papers will be exported into Endnote X9 reference management software (Clarivate Analytics, Philadelphia, PA). After duplicates are removed, the titles and abstracts of studies will be screened against the eligibility criteria and placed into two groups: further review or excluded. The full text of studies classified for further review will be obtained and reviewed again against the eligibility criteria. The reasons for exclusion of studies will be recorded in a PRISMA diagram. Two reviewers will independently complete each step of the process. Any disagreements between the two reviewers will be resolved through discussion. In the instance that a resolution cannot be reached, a third reviewer will be consulted to reach a final decision.

#### Data Extraction

The data extraction table will be designed using the principles of the PRISMA statement for reporting systematic reviews. The items to be extracted from the included papers are study details (ie, authors, year, country of publication, funding, and affiliations), participants (ie, characteristics, flight and simulation details, inclusion and exclusion criteria, attrition, and blinding), intervention and comparator details (ie, intervention, sample size, length of intervention and follow-up, and retention rate), and outcomes (ie, qualitative and quantitative measures of symptoms associated with flight and adverse effects).

### Data Analysis

#### Reporting of Intervention Outcomes

A narrative synthesis of findings structured around the nutrients and herbal compounds investigated will be provided. Tests of heterogeneity between the studies will be conducted using the I^2^ statistic. Meta-analysis by nutrients or supplements will be conducted using Stata software provided there is low heterogeneity (I^2^ value <40%) between two or more studies [16]. The strength of evidence will be judged using the Grading of Recommendations, Assessment, Development, and Evaluation (GRADE) approach [17].

#### Risk of Bias Assessment

Two review authors will independently assess the risk of bias of the included studies using the appropriate Cochrane Collaboration tool (Risk of Bias for Randomized Control Trials II or Robins I for Other Non-Randomized Interventions) [18,19]. The body of evidence from studies with a high risk of bias will be interpreted with caution.

## Results

The scoping review of available functional foods, beverages, and supplements was conducted from March 6, 2019, to August 31, 2019. The systematic literature review commenced on October 1, 2019. The review is expected to be completed in 2020.

## Discussion

There is a lack of valid scientific evidence for the use of foods and nutritional supplements to prevent and manage symptoms and medical conditions arising from long flights. This research employs a combination of both a scoping and traditional systematic literature review to identify the types of products available and examine the evidence surrounding their health claims.

These findings will inform the decision making of commercial airlines, retailers, commercial cabin crews, and the air travelling public around the use of functional foods, beverages, and supplements when flying.

## Abbreviations

CENTRAL: Cochrane Central Register of Controlled Trials
CINAHL: Cumulative Index to Nursing and Allied Health Literature
GRADE: Grading of Recommendations, Assessment, Development and Evaluations
MeSH: Medical Subject Headings
PICOS: participants, interventions, comparisons, outcomes, and study design
PRISMA: Preferred Reporting Items for Systematic Reviews and Meta-Analysis
SIGN: Scottish Intercollegiate Guidelines Network
